# Investigating the role of preference variation in the perceptions of railway passengers in Great Britain

**DOI:** 10.1007/s11116-023-10397-x

**Published:** 2023-06-01

**Authors:** Fredrik Monsuur, Marcus Enoch, Mohammed Quddus, Stuart Meek

**Affiliations:** 1 Transport and Urban Planning Group, School of Architecture, Civil and Building Engineering, Loughborough University, Loughborough, LE11 3TU UK; 2 Centre for Transport Studies, Department of Civil and Environmental Engineering, Imperial College London, London, SW2 2AZ UK; 3 South Western Railway, South Bank Central, 30 Stamford Street, London, SE1 9LQ UK; 4 MaasLab, Energy Institute, University College London, 14 Upper Woburn Place, London, WC1H0NN UK

**Keywords:** Passenger satisfaction, Service quality, Heterogeneity, Latent class model, Ordered logit, Rail transport

## Abstract

This study explores the factors associated with passenger satisfaction on the UK railways. To uncover taste variation, the data was segmented into three homogeneous groups of passengers through a latent class ordered logit model, whereby the class allocation was based on observed personal and trip characteristics. The findings suggest that there is significant variation in the impact of service attributes on overall satisfaction across the segments, ‘class a’, ‘class b’ and ‘class c’. Class a (15% of the sample) consists of moderately dissatisfied to highly dissatisfied passengers, for whom ‘punctuality/reliability’ is most impactful on overall satisfaction. Respondents in this class are much more likely to experience adverse service conditions such as delays or crowding conditions. Class b (32% of the sample) consists of passenger who are quite critical and moderately satisfied, for whom ‘hedonic’ factors such as ‘upkeep and repair of the train’ and ‘seat comfort’ were most impactful. Finally, class c (53% of the sample) consists of passengers that are generally satisfied, and for whom the ‘value for money of the ticket price’ is most impactful on overall satisfaction. Interestingly, for both ‘class b’ and ‘class c’, ‘punctuality/reliability’ plays a more limited role in determining overall satisfaction compared to ‘class a’. This suggests that the role of ‘punctuality/reliability’ in determining overall satisfaction is more complex than presented in the literature thus far. Finally, unobserved taste variation plays an important role in the model, as the class allocation is not always easily linked to observed groups in the data. This paper thus highlights the importance of accounting for unobserved and systematic sources of heterogeneity in the data and could provide useful insights for analysts, policy makers and practitioners, to provide more targeted strategies to improve passenger satisfaction.

## Introduction

Passenger satisfaction is assumed to be an important factor in encouraging a modal shift towards public transport services, and hence deliver economic, social and environmental benefits (de Oña and de Oña 2015). Given that transport operators often operate with limited budgets, it is important to target the most important service attributes, to ensure the success of the service in delivering the benefits associated with public transport services. For this reason, much research has been dedicated to identifying the service attributes that are most important for passengers on public transport services.

Unfortunately, satisfaction with these service attributes is complicated to measure and it is complicated to model their effects on overall passenger satisfaction. One of the reasons for this relates to (unobserved) heterogeneity in the perceptions of passengers. Heterogeneity relates to the fact that passengers have varying preferences, and thus react differently towards the service offered to them. This can be due to a wide range of factors, such as expectations, trip purposes, personal characteristics (e.g., socio-demographics), attitudes and mood. Unfortunately, surveys can only capture a limited set of factors relating to these personal circumstances. This means that potentially important factors remain unobserved by the analyst, whilst it is important to account for this (Jedidi et al. 1997). The literature widely acknowledges the importance of unobserved heterogeneity (Hensher et al. 2010; de Oña et al. 2015; Choi et al. 2021), though most have focused modelling on ‘observed’ heterogeneity. There is thus a need to assess whether unobserved heterogeneity plays a role, and whether this adds to the inferences made by researchers so far.

This paper therefore aims to add to the existing body of literature by investigating the impact of service attributes on overall passenger satisfaction, whilst accounting for observed and unobserved sources of heterogeneity. The data used was obtained from the National Rail Passenger Survey (NRPS). To cater for heterogeneity in the data, a latent class ordered logit model with a class allocation part was adopted. This approach probabilistically segments the data into homogeneous groups through the class allocation part, which utilises observable individual characteristics in the survey data. This approach sheds light on underlying variations in the data that are not observed through the characteristics measured in the survey. The final model specification suggests that railway passengers can be divided in three homogeneous groups, whereby significant variations in the impact of these service attributes are observed across groups. This implies that railway passengers have heterogeneous preferences, and moreover, as not all segments can be easily related to the observed characteristics, that unobserved heterogeneity plays an important role. This suggests that sophisticated modelling approaches are required when studying satisfaction data, with a careful examination of the factors that are related to the variations within the data. Ultimately, such modelling approaches will help clarifying the nature of passenger satisfaction at a deeper understanding and to enable transport operators to more precisely target their customer base.

## Literature review

The marketing literature suggests that if firms achieve to present their customers with a consistent high quality of service, there are able to generate long term profitability (Keiser 1988). Furthermore, it is seen as a predictor of behaviour intentions and consumers choice (Zeithaml 1988; Richard and Allaway 1993; Anderson and Kraus 1981) and thus an important indicator of market share and return on investment (Anderson and Zeithaml 1984; Adam 1994). It is often argued that quality should be measured from the consumers perspective, where perceived quality is the consumers judgement about an entity’s overall excellence or superiority (Zeithaml 1988).

However, in public transport services, performance evaluations of public transport services have traditionally been focused on cost efficiency and effectiveness, as well as supply-oriented measurements of service quality (e.g. average occupancy) which may not always align with what passengers perceive and expect (Rietveld 2005; de Oña and de Oña 2015). Moreover, as most public transport services are operated under a contract with public transport authorities (PTA) and are thus not in competition with other public transport operators, the focus is often to simply comply with the contractual obligations rather than meeting the needs of their passengers, particularly when operating under tight margins set out in contracts. This has often resulted in a decrease of quality, and a lack of quality enhancing investments (Hensher and Stanley 2003).

Over the past two decades, researchers, policy makers and practitioners have therefore aimed to introduce and design a more holistic and customer oriented evaluation of public transport services, which is usually done through customer satisfaction surveys (although a few authors have proposed that ‘Stated Preference’ surveys should be preferred (e.g. Hensher 2015)). The evaluation of ‘passenger satisfaction’ is nowadays part of many public transport contracts (e.g. van de Velde 1999; Hensher and Stanley 2003; Preston and van de Velde 2016; Hirschhorn et al. 2018), where the required survey data on transaction-specific passenger satisfaction is often collected annually or bi-annually by an independent passenger watchdog (e.g. through the ‘National Rail Passenger Survey’ by ‘Transport Focus’ in the UK, or the ‘OV Klantbarometer’ by the ‘CROW’ in the Netherlands). The data obtained from these surveys spurred a wide range of research on the key aspects of passenger satisfaction in public transport services.

### Key aspects of passenger satisfaction

To improve passenger satisfaction, it is important to understand that satisfaction is a multi-attribute construct (e.g. Grönroos 1982; Parasuraman et al. 1985), affected by many factors which may relate to the service delivery itself, as well as socio-economic characteristics, emotional states and individual attitudes towards the service. To capture the multi-attribute nature of satisfaction, it is common practice to measure satisfaction with attributes that describe the service, e.g., ‘cleanliness of the train’, ‘seat comfort’ and so on. These attribute-specific measurements are compared with a measurement of the overall evaluation of the service experience, usually measured through ‘overall satisfaction’ (Anderson et al. 1994). This subsequently enables the analyst to determine how service attributes impact on overall satisfaction. More recently, measures of customer loyalty have also been incorporated in passenger satisfaction surveys.

Regarding the available literature on railway services, most studies consistently conclude that reliability/punctuality is the key service attribute in driving overall satisfaction, whilst other important service attributes include speed, information provision, comfort and cleanliness of the train carriages (Brons and Rietveld 2009; de Oña et al. 2015; Eboli and Mazzulla 2015; Mouwen 2015; Eboli et al. 2018; Grisé and El-Geneidy 2018; Allen et al. 2020). In this sense, the findings for the railway industry are in line with findings across service sectors, where reliability is also found to be the key service attribute (Parasuraman et al. 1985; Johnston 1995; Jun and Cai 2001). For public transport services in general, it is argued that there exists a ‘hierarchy of transit needs’ (based to Maslow’s hierarchy of needs), where reliability is at the bottom of the hierarchy, and is thus the most important service attribute to maintain at all cost (Allen et al. 2019). An interesting aspect to note here is that there seems to be a non-linear relationship between critical incidents, such as poor reliability/punctuality performance, and overall satisfaction (Gijsenberg et al. 2015; Allen et al. 2019a, b, 2020). Despite these findings, evidence from Great Britain’s railways suggests that punctuality/reliability only has a limited impact on railway demand (Wardman and Batley 2021).

### Heterogeneity in satisfaction responses

Whilst findings in the existing literature are relatively consistent, the literature also consistently mentions heterogeneity as a major issue when measuring ‘passenger satisfaction’ (e.g. de Oña and de Oña 2015; Roberts et al. 2021). When the analyst has an a priori belief, e.g., based on theory, that heterogeneity might be associated with certain individual characteristics (e.g., socio-demographics), it is possible to include measurements of these characteristics in surveys to capture this. These observed characteristics can subsequently be used to segment the data into homogeneous groups, after which separate satisfaction models can be estimated, or to be included as covariates to enrich the models. Many potential sources of heterogeneity have been explored and measured in the passenger satisfaction literature, e.g., based on trip purpose, trip frequency, distance travelled and individual characteristics of the respondents, such as attitudes, car ownership, emotions, socio-economic status and so on.

For instance, in the railway related literature, it was found that the views of railway users owning a car significantly deviates from the average railway user, i.e. instead of reliability being the most important service attribute, it was found that travel comfort had a higher weight (Brons et al. 2009). Another study found that some segments, i.e. leisure and student travellers, are mostly price sensitive rather than sensitive to reliability (de Oña et al. 2015). Some of the heterogeneity has also been linked to spatial differences, i.e. passengers in certain regions would react differently to other regions (Eboli et al. 2018; Grisé and El-Geneidy 2018). Other studies used statistical methodologies to correct for observed heterogeneity within one modelling framework. For example, through a ‘SEM-MIMIC’ model, where the model corrects for differences between observed groups/individual characteristics, it was found that comfort is somewhat more important for regular users, and high-income passengers are less satisfied overall. However, reliability is still they key service attribute across passengers (Allen et al. 2020).

Unfortunately, a priori beliefs of the analyst (and the observed characteristics in surveys based on them) might not fully capture the underlying sources of heterogeneity that are present, or there is a lack of homogeneity within the observed group (e.g. Roberts et al. 2021). For instance, if observed characteristics include trip purposes, significant variation within a group could still exist, e.g., a commuter travelling to a crucial work meeting, or to pick up children after work, might place a much higher value on reliability/punctuality compared to other commuters. Heterogeneity of this sort is unobserved as these factors are not measured in surveys (and would increase survey lengths significantly if they were), however they could introduce serious bias in model estimates (Jedidi et al. 1997).

To uncover unobserved heterogeneity, the analyst may apply post hoc segmentation, based on empirical statistical techniques such as cluster analysis or finite mixture/latent class approaches or random parameter approaches. Random parameter approaches have been used in the passenger satisfaction literature (e.g., Hensher et al. 2010; Bordagaray et al. 2014), but may be less suitable for large scale satisfaction datasets. This is because the deviations around the parameter means are not directly linked with observable characteristics, which makes it difficult to infer why these variations take place. Finite mixture and latent class modelling on the other hand relies on a probabilistic classification of respondents and uncovers variation within unique segments that can be identified through observable characteristics of the respondents in the data. This helps the analyst in providing concrete inferences that can be linked to observable groups, which in turn informs policy decisions.

Whilst there is clear merit to these methodologies, there are only a few publications in the passenger satisfaction literature following these approaches, with some focusing on air-rail integration (Yuan et al. 2021), bus rapid transit in Santiago (Chili) and Mexico City (Allen et al. 2019) and metropolitan transport services (Choi et al. 2021). The most insightful finding was obtained from the bus rapid transit study, whereby finite mixture SEM was employed to divide the data into two homogeneous groups, which uncovered significant variations in preferences. Whilst reliability is assumed to be the key factor in passenger satisfaction, it was found that this is not the case for one of the identified groups in Mexico City, which embodied most of the respondents (52%). The same was found for a significant share of respondents in Santiago (46%) (Allen et al. 2019). The authors concluded that users in these segments perceive reliability as fulfilled already, and therefore place a higher value on more ‘hedonic’ attributes of the service. Whilst the latent segments were not identified through observable characteristics in this study, and whilst only a limited set of variables was considered in the segmentation analysis, the findings certainly provide important additional insights and demonstrate that the relationship between overall satisfaction and reliability seems much more complex than initially assumed by the existing literature.

To summarise, limited attention has been given to the issue of unobserved heterogeneity in rail transport services. Recent literature has highlighted the importance of unobserved heterogeneity and uncovered novel insights that could be exploited by policy makers and practitioners to target specific groups of passengers. Therefore, this study contributes to existing knowledge by employing a model that accounts for unobserved heterogeneity through a latent class ordered logit model that considers a large set of attribute specific satisfaction indicators, applied on a nationwide large-scale dataset. Homogeneous groups of passengers with a unique set of preferences are derived, and these groups can be linked to how these respondents currently perceive the service, i.e., attribute specific satisfaction ratings, to improve aspects of the service that are most pressing for individual groups. The findings could contribute to better informed decisions to aim for increases in service quality on the UK railways.

## Data

Survey data was obtained from the National Rail Passenger Survey (NRPS), a paper-based survey which was introduced in the UK rail industry in 1999 by Transport Focus, the independent passenger watchdog in Great Britain. Transport Focus consults around 50,000 railway passengers each year to form a nationwide picture of passenger satisfaction on the British rail network. The outcomes of the NRPS are used as key performance indicators in rail franchises around Great Britain (Transport Focus, 2019). Findings are comparable between survey waves, which allows Train Operating companies (TOCs) to analyse satisfaction trends on their network.

The survey was distributed on railway stations to passengers that were about to board their train, or in some cases on board a train. The surveys are station specific, with the origin station pre-printed on the survey as well as the name of the TOCs calling at that station. Respondents are asked to state their trip purpose, departure time, the TOC they travelled with and the station where they disembarked the train. Then they are asked to provide their satisfaction rating for 30 service attributes, which for instance relate to on-board experience and station experience. Finally, they are asked to provide information on a range of socio-demographic characteristics and trip characteristics. The sampling process for the survey is in most part based on annual passenger numbers, obtained from several sources within the railway industry, such as ticketing databases. A detailed explanation of the sampling process and interview process is provided on the Transport Focus website (Transport Focus, 2020).

For this paper, data from the 37th and 38th survey waves were used, which were conducted from September to November 2017 and from January to March 2018 respectively. The raw dataset consists of 55,183 observations. For the 30 service attributes, respondents can rate their satisfaction on a five-point Likert scale, ranging from ‘very unsatisfied’ to ‘very satisfied’, or to state that they have ‘no opinion’, or to simply leave the question blank. The latter is an unfortunate feature, as regression models cannot deal with missing observations in independent variables.

Because of the non-response issue, two steps were undertaken to generate a dataset that had a useable format for modelling purposes. First, only variables with at least 90% complete responses were selected. Second, observations with missing responses in the retained variables were dropped. After this filtering procedure, 16 attribute specific satisfaction variables, and 32,053 observations were retained, meaning a loss of around 23,000 observations. Whilst this is a sizeable reduction in the sample, the remaining dataset is still very large. The main consequence of the two-step filtering process is that certain variables that might be of importance for particular passenger segments are not taken into account (e.g., variables such as ‘toilet facilities’, ‘satisfaction with connections with other train services’, ‘facilities for car parking’ and ‘shelter facilities at the station’). Finally, service attributes that were too highly correlated (i.e., correlation coefficient of more than 0.8) were discarded as well. One such example is the ‘cleanliness of the inside of the train’ and ‘upkeep and repair of the train’, whereby only the latter was retained. After the data filtering, the attribute specific satisfaction variables were recoded to indicator variables, with value zero for ‘very unsatisfied’, ‘unsatisfied’ and ‘neutral’ and value 1 for ‘satisfied’ and ‘very satisfied’. This step leads to a loss of information, however incorporating categorical variables in a logit model would lead to a large number of additional parameters, affecting model parsimony. We also believe that this large additional effort would yield very limited additional insights.

Table 1 provides information on the attribute specific satisfaction indicators and Table 2 provides information on the socio-demographic variables that were measured by the NRPS. The NRPS also measures overall satisfaction with the journey, through the following question: “Taking into account the station where you boarded the train and the actual train travelled on after being given this questionnaire, how satisfied were you with your journey today?”. This overall satisfaction is also coded on a five point Likert scale ranging from: ‘very unsatisfied’ (2.2%), ‘unsatisfied’ (5%), ‘neutral’ (9.8%), ‘satisfied’(46%) and ‘very satisfied’ (37%), meaning that 83% is at least satisfied with their journey (see Table 1). Considering the attribute specific satisfaction indicators, it is notable that most indicators have positive scores, in particular for the ‘provision of information about train times/platforms’ at the station and the ‘length of time the journey was scheduled to take’. However, for some variables perceptions are quite poor, in particular the ‘availability of seating at the station’ and the ‘value for money for the ticket price’.

**Table 1:** Composition of NRPS sample: Service attribute satisfaction indicators

Attribute specific satisfaction indicators	Mean satisfaction
*Rating of station where train was boarded…*	
Provision of information about train times/platforms	87%
The upkeep/repair of the station buildings/platforms	77%
Cleanliness of the station	80%
The overall station environment	78%
Availability of seating	53%
*Satisfaction with train journey…*	
The frequency of the trains on that route	78%
Punctuality/reliability	77%
The length of time the journey was scheduled to take	85%
The value for money for the price of your ticket	49%
Level of crowding	71%
*Rating of train…*	
Upkeep and repair of the train	76%
The provision of information during the journey	76%
The comfort of the seats	69%
Your personal security whilst on board the train	79%
The cleanliness of the inside of the train	78%
The step or gap between the train and the platform	67%
*Rating of overall journey…*	
Overall satisfaction with the train journey	83%

Regarding the socio-demographics in the sample, summarised in Table 2, it is noteworthy that roughly 40% are commuters (i.e., either daily commuter or non-daily commuter). This means that this group is under sampled, as the share of commuters on the British rail network (roughly 55%) (Department for Transport, 2018). Almost 30% of the respondents in the sample reported experiencing a delay, with 8.4% experiencing a long delay (meaning more than 20 min). Roughly 10% of the respondents did not manage to get a seat during the journey. Finally, most respondents travel on commuter rail services, often around London.

**Table 2:** Composition of NRPS sample: Socio-demographics

Socio demographics	Percent
*Personal characteristics*	
Age below 26	13.0%
Age between 26 and 44	32.3%
Age between 45 and 59	32.9%
Age between 60 and 69	6.3%
Age above 70	2.9%
Age: unknown	12.6%
Gender: Male	44.4%
*Trip purpose and journey characteristics*	
Trip purpose: Personal business	9.6%
Trip purpose: Leisure	36.2%
Trip purpose: Business	12.4%
Trip purpose: Non-daily commute	10.5%
Trip purpose: Daily commute	30.2%
Respondent was standing	10.1%
Respondent experienced a delay	28.0%
Respondent experienced a long delay	8.4%
Respondent travelled during peak time	37.2%
Respondent trusts the TOC	60.1%
*Train service type*	
London Commute	37.9%
non-London commuter rail	15.4%
Long distance rail	20.5%
Inter urban rail	17.0%
Airport rail	5.1%
Local rail	3.9%
*Distance travelled*	
Less than 15 km	25.1%
Between 16 and 50 km	35.2%
Between 51 and 100 km	17.6%
Between 101 and 200 km	11.8%
More than 200 km	10.3%

## Statistical methods

The ordinal nature of the dependent variable - ‘overall satisfaction’ which is coded on a five point Likert scale (i.e. 1 = very unsatisfied, …., 5 = very satisfied), provides a strong motivation for the use of an ordered response model. Methods based on ordinary least squares regression are not appropriate for this type of data because ideally, the outcome of a categorical variable is displayed in terms of probabilities. It is also difficult to describe the ‘satisfaction’ of one individual as being twice as large as that of another individual, thus the model structure imposed on the data is too rigid and will lead to incorrect conclusions being drawn (Greene et al. 2014).

To analyse categorical data with an inherent ordering, McKelvey and Zavoina (1975) proposed to employ an ordered response model, which is extended in this study to a latent class ordered logit model. This model introduces unobserved heterogeneity by probabilistically partitioning the data into several homogeneous classes. To identify these classes, the model uses a ‘class allocation model’, which makes use of systematic heterogeneity captured through exogenous variables to allow the analyst to identify how the population is segmented. The assumption is that the class allocation depends on these exogenous variables. Within each class, the overall satisfaction of the respondent is based on an ordered logit model that considers the influence of all the attribute specific satisfaction indicators.

The latent class ordered logit model formulation can be divided in two steps. First, the latent propensity in the traditional ordered response model is extended by associating the respondent *i* as if he/she belongs to class *s.* The second step is to probabilistically assign the respondent *i* to class *s*. The first step is denoted by the formula below:

1 $${y}_{is}^{\text{*}}= {\beta }_{s}^{{\prime }}{x}_{i}+{\epsilon }_{is}$$

As in the traditional ordered response model, ${y}_{is}^{\text{*}}$ is related through a censoring mechanism to the satisfaction level by the thresholds ${\mu }_{{s}_{j}}$ in the following formula ((Greene et al. 2014a, b):

2 $${y}_{i}=j \Leftarrow {{\mu }_{s}}_{j-1}<{y}_{i}^{\text{*}}\le {\mu }_{{s}_{j}}$$

As in the standard ordered response model, the model contains unknown marginal utilities $\beta$ and threshold parameters ${\mu }_{{s}_{j}}$ where (${\mu }_{{s}_{0}}=-\infty$ and ${\mu }_{{s}_{J}}=\infty$). The assumptions regarding the error term ${\epsilon }_{is}$ includes independence from ${x}_{i}$, distributed across individuals *i* and segments *s*. The resulting conditional probability, in which *F* represents the logistic distribution, can be written as follows:

3 $${P}_{i}\left(\left(j\right)\right|s)= F\left({\mu }_{{s}_{j}}-{\beta }_{s}^{{\prime }}{x}_{i}\right)-F\left({\mu }_{{s}_{j-1}}-{\beta }_{s}^{{\prime }}{x}_{i}\right)$$

The second step is to formulate the class allocation model part of the latent class ordered logit model. The multinomial logit model is used for the class allocation model to probabilistically allocate the satisfaction rating of individual *i* to class *s* (Eluru et al. 2012). The random utility for allocating an individual *i* to class *s* is described as follows:

4 $${U}_{is}^{*}={\alpha }_{s}^{{\prime }}{\gamma }_{i}+{\xi }_{is}$$

The model contains marginal utilities ${\alpha }_{s}^{{\prime }}$ associated with independent variables representing the respondents’ characteristics, described by ${\gamma }_{i}$. Finally, ${\xi }_{is}$ is a random error term and assumptions include independence from ${\gamma }_{i}$, distributed across individuals *i* and classes *s*. The probability that the satisfaction rating associated with respondent *i* belongs to classes *s* is as follows:

5 $${P}_{is}= \frac{\text{e}\text{x}\text{p}\left({\alpha }_{s}^{{\prime }}{y}_{j}\right)}{\sum _{s}\text{e}\text{x}\text{p}\left({\alpha }_{s}^{{\prime }}{\gamma }_{j}\right)}$$

The formulations in step one and step two can be combined to obtain the unconditional probabilities for the latent class ordered logit model specification. The unconditional probability of respondent *i* rating the service with satisfaction level *j* is given as follows (Eluru et al. 2012):

6 $${P}_{i}\left(j\right)= \sum _{s=1}^{S}{P}_{i}\left(\right(j\left)\right|s){P}_{is}$$

The parameters that need to be estimated for the model are ${\beta }_{s}$ and ${\mu }_{{s}_{j}}$ relating to the latent propensity model part, and ${\alpha }_{s}$, relating to the class allocation model part, for each class *s* and the number of classes *S*. The log-likelihood function that needs to be maximised for the entire dataset is as follows:

7 $$L= \sum _{i=1}^{I}\text{l}\text{o}\text{g}\left({P}_{i}(j\right))$$

The modelling approach usually starts with a model that considers two classes. Then for each model step, classes are added until further additions do not yield a superior model fit or intuitive interpretation (or when the model simply does not converge properly). As the models are non-nested, the models can be compared by the Akaike Information Criterion (AIC) or the Bayesian Information Criterion (BIC) to identify which model is most suitable. The BIC is preferable, as it accounts for the number of observations present in the data, while the AIC generally favours models with more segments for large data samples (Bhat 1997). The BIC is formulated as follows:

$$-2\text{ln}\left(L\right)+k\text{l}\text{n}\left(n\right)$$

where $\text{ln}\left(L\right)$ is the log-likelihood at convergence, $k$ the number of parameters and $n$ the number of observations. Estimation is terminated at the point where the lowest value of BIC is reached. The models were estimated using the ‘Apollo’ package in R (Hess and Palma 2019).

## Results

Four different models have been considered for this paper, including:

- Model 1: the traditional ordered logit model,
- Model 2: a latent class ordered logit model with two classes,
- Model 3: a latent class ordered logit model with three classes and.
- Model 4: a latent class ordered logit model with four classes.

The model specifications resulted from a systematic process of selecting suitable candidate variables, combining variables, and removing statistically insignificant variables. This process was guided by prior research, intuitiveness, and extensive analysis of summary statistics. Several models on the identified classes in the data and observable segments (e.g., based on trip purpose) were formulated to validate the results.

Model 4 disappointingly (as four classes is not too many for a large sample size) did not yield stable results and was thus discarded. To compare model fit between the remaining models, the Bayesian Information Criterion (BIC) was used, whereby the model with the lowest BIC value is preferred. The BIC values for the final specifications of model 1, model 2 and model 3 are 54,717, 54,101 and 53,962 respectively. The BIC statistics confirm that model 3 offers superior data fit relative to model 1 and model 2. This indicates that passenger satisfaction can be best examined through the segmentation of the sample into three classes. In the following section, the focus is therefore only on model 3, the latent class ordered logit model with three classes.

Before discussing the impact of the model parameters on class segmentation and overall passenger satisfaction, it is important to first provide an intuitive interpretation of the model. The model itself consists of two components: a class allocation model component, and a satisfaction component. The class allocation component allocates a class-specific probability to each respondent, which is influenced by observed individual characteristics related to the respondent. These individual characteristics are included as covariates in the class allocation model component. This class allocation part is related to the satisfaction component of the model, which provides a class-specific parameter estimate for each satisfaction attribute. Each class essentially represents a homogeneous group of respondents, and the impact of satisfaction attributes will vary between these groups. The resulting model introduces taste variation, mapped to a set of homogenous groups with observable and unobservable characteristics.

### Interpretation of the results

The modelling results are presented in Table 3. This section discusses the findings in three steps:

1. Class allocation part of the model.
2. Satisfaction component of the model.
3. Marginal effects.

**Table 3:** Modelling results

	Class a	Class b	Class c
**Class allocation model component (class c reference category)**			
*Mean class probability*	*0.15*	*0.32*	*0.53*
Alternative specific constant	-2.082	-0.476	
Trip purpose: business	-0.402		
Trip purpose: personal business	-0.765	-0.490	
Trip purpose: Leisure	-0.860	-0.795	
Respondent was standing	1.882	0.432	
Respondent experienced a delay	1.524	1.107	
Respondent experienced a severe delay	2.074		
Respondent travelled during peak time	0.199		
London Commute	-0.423		
non-London commuter rail		0.346	
Respondent travelled 0–25 km by train		-0.279	
Respondent travelled 50-100 km by train	0.331		
Respondent travelled 100–200 km by train	0.285		
**Satisfaction rating model component**			
*Rating of the station where the train was boarded…*			
Provision of information about train times/platforms	0.565	0.968	0.536
The upkeep/repair of the station buildings/platforms		0.765	0.338
The overall station environment	0.479	0.742	0.672
Availability of seating		0.491	0.571
*Satisfaction with the train journey…*			
The frequency of the trains on that route	0.932	1.062	0.540
Punctuality/reliability	1.179	1.099	0.823
The length of time the journey was scheduled to take	0.480	1.140	0.734
The value for money for the price of your ticket	0.563	0.926	1.002
Level of crowding	0.565	0.977	0.777
*Rating of the train…*			
Upkeep and repair of the train	0.364	1.328	0.892
The provision of information during the journey	0.541	0.888	0.648
The comfort of the seats	0.481	1.337	0.646
Your personal security whilst on board the train		0.665	0.256
The step or gap between the train and the platform		0.286	0.341
*Thresholds*			
Threshold 1 (‘very unsatisfied’ to ‘unsatisfied’)	1.036	-1.219	-7.744
Threshold 2 (‘unsatisfied’ to ‘neutral’)	3.028	2.738	-6.545
Threshold 3 (‘neutral’ to ‘satisfied’)	3.914	6.465	1.858
Threshold 4 (‘satisfied’ to ‘very satisfied’)	7.028	12.064	6.689
LL(final, whole model)	-26,628		
AIC	53,392		
BIC	53,962		
Number of observations	32,053		

* only variables significant at the 5% level are included in the model

### Class allocation model component

**Fig. 1:**
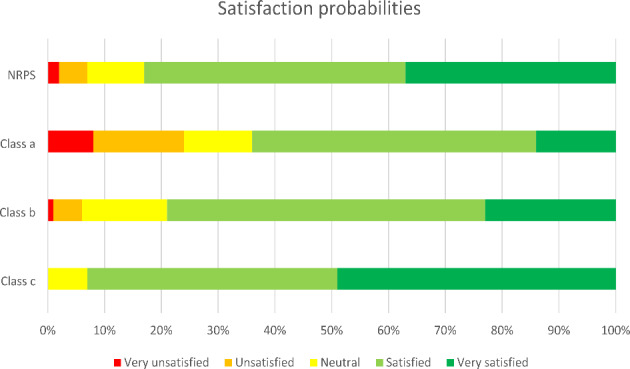
Average class-specific probabilities and NRPS probability for overall satisfaction rating

The model consists of three classes as shown in Table 3, where ‘class c’ is the reference category. Coefficients presented in the table thus indicate the propensity for being ‘class a’ or ‘class b’ relative to ‘class c’. The class-specific results are mapped to the observable journey and trip characteristics of the respondent, which include factors such as the trip purpose of the respondent, circumstances regarding the service delivery (e.g., delays or unavailable seats), type of rail service used, distance travelled and the time of travel. For interpretation purposes, the class-specific posterior mean probabilities were calculated for each covariate included in the class allocation model and presented in Table 4. Finally, Fig. 1 presents the ‘overall satisfaction’ levels for each class, contrasted with the overall satisfaction levels across the NRPS sample.

**Table 4:** Posterior mean class-specific probabilities (Please note, coefficients are made bold when the probability is higher than the mean class probability)

Class allocation component	P(class a)	P(class b)	P(class c)
Mean probability	0.15	0.32	0.53
Trip purpose: business	0.12	0.39	0.49
Trip purpose: personal business	0.12	0.30	0.58
Trip purpose: Leisure	0.10	0.24	0.66
Trip purpose: Daily commute (base)	0.21	0.36	0.43
Trip purpose: Non-daily commute (base)	0.17	0.37	0.46
Respondent was standing	0.43	0.29	0.28
Respondent was seated (base)	0.12	0.32	0.57
Respondent did not experience a delay (base)	0.07	0.29	0.64
Respondent experienced a delay	0.33	0.39	0.27
Respondent experienced a severe delay	0.63	0.21	0.16
Respondent travelled off peak time (base)	0.12	0.31	0.57
Respondent travelled during peak time	0.19	0.33	0.47
London Commute	0.14	0.32	0.54
non-London commuter rail	0.15	0.36	0.49
Respondent travelled 0–25 km by train	0.15	0.31	0.54
Respondent travelled 50-100 km by train	0.15	0.33	0.52
Respondent travelled 100–200 km by train	0.15	0.32	0.53

‘Class a’ is mainly characterised by respondents that encountered adverse service conditions, which caused them to be delayed (e.g., in case of a severe delay, the class-specific probability is 0.66) and/or had to stand during the journey. The average class-specific probability is 0.15, which indicates that a minor group of respondents is allocated to this class. Based on Fig. 1 (and the threshold boundary parameters in Table 3), it can be concluded that they are generally more likely to be dissatisfied with the service provision. The behavioural interpretation for this is obvious, as these respondents encountered severe issues during their journey. It should be noted that Fig. 1 underappreciates that there is a group of participants with a very high class allocation probability for ‘class a’, which are generally very negative (e.g., if the class allocation probability is above 0.6, which is the case for 2,379 respondents, 71% of them rates their overall satisfaction as ‘very unsatisfied’ or ‘unsatisfied’).

‘Class b’ is not easily characterised by any of the observed individual characteristics, though business and commuters are somewhat more likely to be part of this segment, relative to the mean-class probabilities. There is also an increased probability that respondents in this segment encounter a delay, however the deviation from the mean probability across the sample is limited, whilst respondents experiencing a severe delay are less likely to be allocated to ‘class b’. The most meaningful characterisation of respondents in ‘class b’ can be derived from the ‘overall satisfaction’ ratings they provide, where it is notable that they are not likely to be unsatisfied, but not likely to give a very high rating for the service either (see Fig. 1). As for ‘class a’, it should be noted that for high class allocation probabilities for ‘class b’, respondents become more negative about the service provision (e.g., if the class allocation probability for ‘class b’ is above 0.5, which is the case for 6,166 participants, only 3% of them rate their overall satisfaction as ‘very satisfied’, 58% as ‘satisfied’ and 30% as ‘neutral’). A behavioural interpretation could be that respondents allocated to ‘class b’ have higher expectations than other segments and are thus less likely to rate the service as ‘very satisfied’. These higher expectations could be linked with individual characteristics that are not measured in the survey, such as income levels, socio-economic status, education level and so on. The average class-specific probability is 0.32, which indicates that a significant number of travellers is represented by this segment.

‘Class c’ is mainly characterised by personal business and leisure-oriented travellers, often travelling off-peak and generally not encountering any adverse service conditions (though, there is still a significant probability that commuters fall into this class). They are usually satisfied with the railway service and are likely to provide high ratings to the service (see Fig. 1). For this class, higher class allocation probabilities go hand in hand with higher overall satisfaction ratings (e.g., if the class allocation probability is above 0.6, which is the case for 15,367 respondents, 69% of them rates their overall satisfaction as ‘very satisfied’). This could be because respondents in this class may feel that their expectations of the railway service are either met or perhaps even exceeded. The average class-specific probability is 0.53, which means that class c represents most travellers on the railway network.

Finally, some covariates have a major impact on class allocation (e.g., adverse service conditions, and leisure and personal business trip purposes). However, most covariates do not have a major impact on class allocation, with even trip purposes such as commuting having a limited impact. It may be the case that unobserved factors such as income levels, or occupation play an underlying role (and if measured, a simple segmentation might suffice to uncover these taste variations present in the data).

### Satisfaction rating model component

The class allocation model divides the satisfaction rating component into three homogeneous classes, where satisfaction attributes have a unique effect for each class. The satisfaction attributes included in this model are related to attributes that describe the station, the journey and the respondent’s train, and the coefficients represent the impact of the satisfaction attribute on overall satisfaction for when the respondent is ‘satisfied’ or ‘very satisfied’ with the service attribute. The findings for each class are discussed in the following subsections. Table 3 provides the estimated coefficients, and to contextualise the findings, Table 5 provides the average satisfaction ratings for each service attribute per class (these are somewhat crudely calculated by allocating each individual to the class for which it has the highest probability, after which the average satisfaction was calculated for each class).

**Table 5:** Percentage of respondents satisfied with service attribute

Percentage satisfied per attribute			
**Respondent is satisfied with**:	Class a	Class b	Class c
*Rating of the station where the train was boarded…*			
Provision of information about train times/platforms	78%	85%	89%
The upkeep/repair of the station buildings/platforms	69%	74%	79%
The overall station environment	70%	76%	80%
Availability of seating	42%	51%	56%
*Satisfaction with the train journey…*			
The frequency of the trains on that route	60%	75%	82%
Punctuality/reliability	40%	68%	86%
The length of time the journey was scheduled to take	67%	81%	89%
The value for money for the price of your ticket	31%	42%	54%
Level of crowding	46%	67%	76%
*Rating of the train…*			
Upkeep and repair of the train	67%	73%	79%
The provision of information during the journey	66%	73%	79%
The comfort of the seats	60%	65%	72%
Your personal security whilst on board the train	72%	76%	81%
The step or gap between the train and the platform	61%	66%	69%

### Satisfaction rating model component: ‘Class a’

Respondents in ‘class a’ are more likely to have encountered a significant service failure. Regarding the station attributes, only two variables were statistically significant, i.e., ‘provision of information’ and ‘overall station environment’. The impact is low, which indicates they do not have a large impact on overall satisfaction.

Considering the ‘satisfaction with the train journey’ indicators, the satisfaction attribute ‘punctuality/reliability’ has a major impact and compared to the other two classes. This finding is obvious given that these respondents likely encountered adverse service conditions. Aside from this, the ‘frequency of the trains on the route’ also has a high impact. However, the other three satisfaction attributes, i.e., ‘length of time the journey was scheduled to take’, ‘value for money for the ticket price’ and ‘level of crowding’ have a much lower impact compared to the other classes. The low impact for ‘level of crowding’ is rather curious, as ‘class a’ has a higher probability for respondents that were unable to obtain a seat during their journey. It could thus be that even proper seat provision will not significantly increase their satisfaction when their train is late.

Regarding the ‘satisfaction with the train’ indicators, only three factors are statistically significant and with a very low impact compared to ‘class b’ and ‘class c’.

Finally, Table 5 provides the percentage satisfied per service attribute. It is noticeable that the satisfaction with any service attribute is significantly lower than for ‘class b’ and ‘class c’ and for ‘punctuality/reliability’, ‘level of crowding’ and ‘the value for money for the price of your ticket’. Thus, the overall satisfaction rating of respondents in ‘class a’ are mainly driven by the ‘punctuality/reliability’ of the train. Respondents are unsatisfied with this service attribute, as well as the ‘level of crowding’ and ‘the value for money’, and with the railway service in general.

### Satisfaction rating model component: ‘Class b’

Respondents in ‘class b’ consist of more critical passengers, who may feel that their expectations have not been fully met. Respondents that travel for business or commuting purposes are somewhat more likely to be part of this class, though no single trip purpose is obviously related to ‘class b’.

Considering the service attributes that describe the railway station, it appears that satisfaction with these attributes have a higher impact on overall satisfaction compared to ‘class a’ and ‘class c’. This is evidenced by the impact of satisfaction with ‘provision of information’, ‘upkeep/repair of the station buildings/platforms’ and the ‘overall station environment’. This could be in line with the suggestion that respondents in this class are somewhat more critical, perhaps have higher expectations from the railway service, and pick up on aspects of the service that are not generally considered as core requirements.

Regarding the ‘satisfaction with the train journey’ indicators, it is notable that all service attributes in this category have a high impact. The impact of ‘frequency of trains’ and especially ‘length of time the journey was scheduled to take’ is much higher for ‘class b’ relative to class c. This suggests a preference for fast and frequent train services, perhaps due to a higher value of time. The ‘value for money for the price of your ticket’ also has a high impact relative to ‘class a’. Perhaps more notable is the high impact on ‘level of crowding’ compared to the other two classes, which suggests an aversion of busy trains.

Considering the ‘satisfaction with the train’ indicators, it seems that these are of major importance for ‘class b’, compared to the other two classes. Particularly the impact of ‘upkeep and repair of the train’ and ‘the comfort of seats’ stand out, suggesting that satisfaction with these factors is a major driver of overall satisfaction. This is a finding that is important for train operators, as there has been frequent criticism of new rolling stock regarding the quality of the seating, even described as ‘ironing boards’ (The Times, 2021).

Finally, Table 5 provides the percentage satisfied per service attribute. It is noticeable that satisfaction with any service attribute is somewhat lower compared to ‘class c’ which reinforces the conclusion that respondents in ‘class b’ may have higher expectations and thus are more critical of the railway services. The most noticeable difference with ‘class c’ is found on satisfaction with ‘punctuality/reliability’, which also reinforces the conclusion that respondents allocated to this class might have a higher value of time. Thus, respondents in ‘class b’ place a high value on fast and frequent services, as well as comfortable trains.

### Satisfaction rating model component: ‘Class c’

Respondents in ‘class c’ consist of respondents more likely to be ‘business’, ‘personal business’ or ‘leisure’ travellers. They are generally ‘satisfied’ or ‘very satisfied’ with the railway service. Regarding the attributes that describe the railway station, the ‘overall station environment’ seems to be important. A behavioural explanation could be that they place a high value on the amenities that railway stations have on offer.

Regarding the satisfaction with the train journey, it is quite notable that ‘frequency’ and ‘punctuality’ have a lower impact. The reason for this could be twofold: first, respondents in this category are unlikely to suffer delays, second, respondents may have a lower value of time. The most important aspect for this group of passengers seems to be the ‘value for money’ of the ticket price, which indicates that they are price sensitive and could benefit from targeted pricing policies.

Regarding attributes that describe the train, it is noteworthy that these aspects seem to have a lower impact on the overall satisfaction compared to ‘class b’, but a higher impact compared to ‘class a’. This could be because they might have somewhat lower expectations from the service compared to ‘class b’, and/or are already largely satisfied with the service provision as it is. The latter possibility is certainly in line with the findings in Table 5, where satisfaction ratings for train related aspects for this class are relatively high compared to the two other classes.

Concluding, respondents in ‘class c’ are likely satisfied already, and the only aspect that could have a material impact in further improving their satisfaction levels would be to improve the ‘value for money for the ticket price’. Given that only 54% in this class is satisfied with the ticket price (see Table 5), there is still scope for improvements. The question is of course whether this is commercially viable, as the data section already indicates that more than half of the leisure passengers, which are more likely to be part of ‘class c’, already travel on off-peak tickets often booked in advance, which are amongst the cheapest tickets available on the network.

### Marginal effects

Table 6 provides the marginal effects derived from the modelling results, which provide an intuitive oversight of the average effects of the variables across the data (Tables 7, 8 and 9 provide this for the class specific marginal effects whereby the heterogeneity between classes is visible). The satisfaction attributes are indicator variables; therefore, one simply calculates the probability for each satisfaction outcome for when the satisfaction attribute has value ‘1’ (i.e., ‘very satisfied’ and ‘satisfied’) and value ‘0’ (i.e., ‘neutral, ‘unsatisfied’ and ‘very unsatisfied’), holding all else constant. The probability for when the satisfaction attribute has value ‘1’ is then subtracted from the probability when the satisfaction attribute has value ‘0’ and divided by the probability when the satisfaction attribute is ‘0’ through the following formula:

8 $$\frac{\partial {y}_{i}}{{\partial X}_{ijn}}=\frac{P\left({y}_{i}>j if {X}_{ijn}=1\right)-P\left({y}_{i}>j if {X}_{ijn}=0\right) }{P\left({y}_{i}>j if {X}_{ijn}=0\right)}$$

**Table 6:** Marginal effects derived from modelling results

	Very unsatisfied	Unsatisfied	Neutral	Satisfied	Very satisfied
*Rating of station where train was boarded…*					
Provision of information about train times/platforms	-0.31	-0.20	-0.20	-0.08	0.28
The upkeep/repair of the station buildings/platforms	-0.04	-0.10	-0.15	-0.07	0.17
The overall station environment	-0.27	-0.16	-0.20	-0.10	0.30
Availability of seating	-0.03	-0.06	-0.15	-0.10	0.20
*Satisfaction with train journey…*					
The frequency of the trains on that route	-0.47	-0.28	-0.22	-0.07	0.31
Punctuality/reliability	-0.56	-0.34	-0.28	-0.09	0.45
The length of time the journey was scheduled to take	-0.29	-0.22	-0.25	-0.10	0.38
The value for money for the price of your ticket	-0.32	-0.21	-0.27	-0.16	0.45
Level of crowding	-0.32	-0.21	-0.25	-0.11	0.38
*Rating of train…*					
Upkeep and repair of the train	-0.24	-0.22	-0.30	-0.13	0.48
The provision of information during the journey	-0.31	-0.19	-0.21	-0.09	0.31
The comfort of the seats	-0.29	-0.24	-0.27	-0.10	0.37
Your personal security whilst on board the train	-0.13	-0.15	-0.18	-0.11	0.32
The step or gap between the train and the platform	-0.02	-0.04	-0.09	-0.06	0.11

**Table 7:** Marginal effects derived from modelling results for class a

	Very unsatisfied	Unsatisfied	Neutral	Satisfied	Very satisfied
*Rating of station where train was boarded…*					
Provision of information about train times/platforms (class a)	-0.30	-0.20	-0.14	0.09	0.56
The upkeep/repair of the station buildings/platforms (class a)	n/a	n/a	n/a	n/a	n/a
The overall station environment (class a)	-0.22	-0.16	-0.14	0.06	0.50
Availability of seating (class a)	n/a	n/a	n/a	n/a	n/a
*Satisfaction with train journey…*					
The frequency of the trains on that route (class a)	-0.55	-0.32	-0.20	0.18	1.14
Punctuality/reliability (class a)	-0.55	-0.44	-0.25	0.29	2.00
The length of time the journey was scheduled to take (class a)	-0.22	-0.16	-0.14	0.06	0.40
The value for money for the price of your ticket (class a)	-0.33	-0.22	-0.15	0.06	0.60
Level of crowding (class a)	-0.33	-0.21	-0.14	0.08	0.67
*Rating of train…*					
Upkeep and repair of the train (class a)	-0.22	-0.11	-0.08	0.04	0.27
The provision of information during the journey (class a)	-0.22	-0.21	-0.14	0.08	0.67
The comfort of the seats (class a)	-0.33	-0.21	-0.08	0.06	0.50
Your personal security whilst on board the train (class a)	n/a	n/a	n/a	n/a	n/a
The step or gap between the train and the platform (class a)	n/a	n/a	n/a	n/a	n/a

* Please note that the n/a’s are generated for variables that were not statistically significant for class a. The coefficients in this table can be multiplied by 100 to obtain a percentage change effect

**Table 8:** Marginal effects derived from modelling results for class b

	Very unsatisfied	Unsatisfied	Neutral	Satisfied	Very satisfied
*Rating of station where train was boarded…*					
Provision of information about train times/platforms (class b)	-0.62	-0.33	-0.22	-0.07	0.71
The upkeep/repair of the station buildings/platforms (class b)	-0.54	-0.33	-0.18	-0.07	0.56
The overall station environment (class b)	-0.48	-0.33	-0.18	-0.07	0.50
Availability of seating (class b)	-0.35	-0.20	-0.07	-0.05	0.30
*Satisfaction with train journey…*					
The frequency of the trains on that route (class b)	-0.62	-0.33	-0.22	-0.06	0.92
Punctuality/reliability (class b)	-0.63	-0.33	-0.22	-0.06	0.92
The length of time the journey was scheduled to take (class b)	-0.65	-0.33	-0.26	-0.08	1.00
The value for money for the price of your ticket (class b)	-0.56	-0.20	-0.25	-0.10	0.75
Level of crowding (class b)	-0.59	-0.33	-0.24	-0.08	0.79
*Rating of train…*					
Upkeep and repair of the train (class b)	-0.70	-0.50	-0.32	-0.09	1.27
The provision of information during the journey (class b)	-0.55	-0.33	-0.18	-0.08	0.67
The comfort of the seats (class b)	-0.71	-0.50	-0.28	-0.08	1.36
Your personal security whilst on board the train (class b)	-0.45	-0.33	-0.18	-0.05	0.41
The step or gap between the train and the platform (class b)	-0.22	0.00	-0.07	-0.03	0.14

* The coefficients in this table can be multiplied by 100 to obtain a percentage change effect

**Table 9:** Marginal effects derived from modelling results for class c

	Very unsatisfied	Unsatisfied	Neutral	Satisfied	Very satisfied
*Rating of station where train was boarded…*					
Provision of information about train times/platforms (class c)	-0.41	-0.41	-0.22	-0.12	0.20
The upkeep/repair of the station buildings/platforms (class c)	-0.29	-0.29	-0.13	-0.08	0.11
The overall station environment (class c)	-0.49	-0.49	-0.33	-0.14	0.25
Availability of seating (class c)	-0.44	-0.43	-0.25	-0.14	0.18
*Satisfaction with train journey…*					
The frequency of the trains on that route (class c)	-0.42	-0.42	-0.25	-0.12	0.19
Punctuality/reliability (class c)	-0.56	-0.56	-0.44	-0.17	0.34
The length of time the journey was scheduled to take (class c)	-0.52	-0.52	-0.33	-0.15	0.32
The value for money for the price of your ticket (class c)	-0.63	-0.63	-0.50	-0.23	0.40
Level of crowding (class c)	-0.54	-0.54	-0.44	-0.17	0.31
*Rating of train…*					
Upkeep and repair of the train (class c)	-0.59	-0.59	-0.44	-0.19	0.38
The provision of information during the journey (class c)	-0.48	-0.48	-0.33	-0.14	0.25
The comfort of the seats (class c)	-0.48	-0.48	-0.25	-0.16	0.24
Your personal security whilst on board the train (class c)	-0.23	-0.23	-0.13	-0.06	0.09
The step or gap between the train and the platform (class c)	-0.29	-0.29	-0.25	-0.06	0.11

* The coefficients in this table can be multiplied by 100 to obtain a percentage change effect

where ${X}_{ijn}$ represents the *n*th explanatory variable associated with response *j* for respondent *i*. The marginal effects are useful for predicting the impact that certain improvements on the network could have on passenger satisfaction.

When multiplied by 100, the numbers in Table 6 can be interpreted as the percentage change in the probability of a satisfaction outcome due to a change in the independent variable from ‘0’ to ‘1’, holding all else constant. For instance, the table indicates that if the respondent is satisfied with ‘Level of crowding’, the probability for rating the overall passenger journey as ‘very satisfied’ is on average 38% higher than if the respondent is not satisfied with ‘Level of crowding’. Generally, the variables with the largest decrease in probability on the ‘very unsatisfied’ and ‘unsatisfied’ category, as well as large increases on the ‘satisfied’ and ‘very satisfied’ categories can be seen as core aspects of the service, whilst variables that only have a large increase in probability for the ‘very satisfied’ category can be seen as more ‘hedonic’ aspects of the service.

Given this interpretation, satisfaction with service attributes related to the journey have, on average, a very significant influence on overall passenger satisfaction. The variable with the most consistently large impact across all overall passenger satisfaction outcomes is ‘Punctuality/reliability’. This even though the variable is only most impactful for a small segment (class a). This suggest that the negative response to unreliable railway performance is so extreme, that this influences the average effects throughout the dataset. This again highlights the importance of accounting for taste variation, as if unaccounted for, the analyst could erroneously believe that reliability/punctuality is most impactful for a randomly selected respondent and could underappreciate other aspects of the service. What can be concluded is thus that unreliable performance leads to extreme responses, which indicates that it is a core aspect for passengers. If railway companies do provide a punctual service, more ‘hedonic’ factors start to play a role, and then it is important to focus on these ‘hedonic’ factors to further improve satisfaction. In this sense, the findings derived from the marginal effects strengthen the conclusion from previous literature (e.g., Allen et al. 2019) that reliability is a core aspect that needs to be satisfied first before attention can be given to more ‘hedonic’ aspects, such as the ‘value for money for the price of your ticket’, ‘upkeep and repair of the train’, ‘comfort of the seats’ and ‘information provision during the journey’. Based on the class specific results, it can be concluded however, that for most respondents, the core aspect of the service is satisfactory, and that railway companies may focus on these more ‘hedonic’ aspects to increase passenger satisfaction, and hopefully, subsequently increase demand for railway services.

## Discussion

This study explored the determinants of passenger satisfaction in the context of Great Britain’s railways, through a large nationwide dataset (NRPS), whilst accounting for observed and unobserved sources of taste variation. This was done through a latent class ordered logit model, whereby these homogeneous groups were derived based on observed characteristics related to the trip purpose of the respondent, as well as circumstances regarding the service delivery. The results indicate that railway passengers can be segmented into three homogeneous groups and significant differences in the impact of service attributes are observed between the segments, in particular regarding the impact of ‘punctuality/reliability’.

Specifically, ‘class a’, which has a class allocation probability of 15%, consists of passengers that are likely travelling on delayed and/or overcrowded trains. Punctuality/reliability has the highest impact on overall satisfaction for this segment. Respondents are unsatisfied with this service attribute, as well as the ‘level of crowding’ and ‘the value for money’, and with the railway service in general. These findings are in line with literature considering ‘critical incidents’ or poor operational performance, whereby service failures have a major impact on overall satisfaction (Gijsenberg et al. 2015; Allen et al. 2019a, b, 2020).

For ‘class b’, which has a class allocation probability of 32%, it can be concluded that the main impact on overall satisfaction relates to ‘hedonic’ attributes such as ‘the comfort of the seats’ and ‘upkeep and repair of the train’. They also place a high value on fast and frequent services, which could indicate a high value of time. In general, they are not dissatisfied, but not likely to be ‘very satisfied’ either. This could indicate that they have higher expectations compared to the other segments. In general, there is no obvious covariate in the class-allocation model that determines class membership for ‘class b’, though business and commuters are somewhat more likely to be part of this segment. One can speculate that unobserved factors, not measured in the survey, such as income, socio-economic status and education level could be determining factors.

Finally, for ‘class c’, which has a class allocation probability of 53%, it can be concluded that the main impact on overall satisfaction is ‘the value for money of the ticket price’ and respondents allocated to this class are highly satisfied with the railway service in general. This could be because they may feel that their expectations of the railway service are either met or perhaps even exceeded. Respondents in this class are mainly characterised by personal business and leisure-oriented travellers, often travelling off-peak and generally not encountering any adverse service conditions (though, there is still a significant probability that commuters fall into this class).

Whilst individual characteristics such as trip purpose do impact on segments (and in the case of leisure significantly impact), they alone do not uncover the variation observed in the data. Indeed, one of the segments, ‘class b’, is not obviously defined by any of these observed characteristics in the data. Moreover, a major share of the railway market, commuters, whilst being more likely to be part of ‘class a’ and ‘class b’, are not a homogeneous group, but have significant variations in their preferences. Unfortunately, we can only speculate as to why this variation occurs (e.g., as mentioned, perhaps income status, socio-economic status, education levels play an important role).

The most interesting finding from the model concerns the varying impact of ‘punctuality/reliability’, which according to the literature is the main factor impacting on passenger satisfaction (e.g., Brons and Rietveld 2009; de Oña et al. 2015; Eboli and Mazzulla 2015; Mouwen 2015; Eboli et al. 2018; Grisé and El-Geneidy 2018; Allen et al. 2020). This study demonstrates that the relationship is more complex, whereby ‘punctuality/reliability is the main factor for the smallest of the three identified segments only. For the other two segments, ‘hedonic’ factors play a more important role. Considering the marginal effects, it can be seen that ‘punctuality/reliability’ still has a strong underlying role across the whole sample, as marginal impacts on ‘very unsatisfied’ and ‘unsatisfied’ categories are high. Thus, to ensure respondents improve their perception from ‘unsatisfied’ to ‘satisfied’, punctuality/reliability improvements are very likely to have a major impact. However, to ensure that respondents go from being ‘neutral’ to ‘satisfied’, or from ‘satisfied’ to ‘very satisfied’, improvements in more ‘hedonic’ factors, related to comfort and so on might be more important, which is in line with recent findings obtained by Allen, Muñoz and OrtúzaAllen et al. (2019a, b). Interestingly, this finding may also be linked with evidence from Great Britain’s railways, whereby it is suggested that punctuality/reliability only has a limited impact on railway demand (Batley et al. 2011; Wardman and Batley 2021). It could be that improvements on ‘hedonic’ service attributes have a higher impact on attracting demand, as they may target potential customers with higher requirements and expectations from railway services.

### Policy implications

Some policy implications can be derived from the findings of this study. The segmentation approach utilised in this study could help railway operators to better understand the customers they serve and derive more targeted customer-oriented strategies to improve passenger satisfaction and attract more demand.

Specifically, for ‘class b’, more attention should be provided to ‘hedonic’ service attributes, such as the ‘comfort of the seats’, the ‘upkeep and repair of the train’. Indeed, many new fleets on Britain’s rail network attracted severe criticism regarding their comfort levels (The Times, 2021). Railway operators should account for this factor in future rolling stock procurement, or whenever existing rolling stock needs refurbishment. Indeed, given the limited demand impact of ‘punctuality/reliability on demand, (Batley et al. 2011; Wardman and Batley 2021), it might be that these ‘hedonic’ factors may have been underappreciated on Britain’s railways and could play a role in attracting demand. In other words, meeting the core requirements, a punctual railway, is not sufficient.

More attention should also be given to the handling of adverse service conditions. The findings regarding ‘class a’ demonstrate that passengers respond extremely negative to severe delays and overcrowding. Whilst it is impossible to avoid these issues from happening, perhaps there are more targeted measures could alleviate the poor service experienced by customers. Previous literature for instance suggests that more targeted information provision, whereby passengers are timely informed, such that they can make alternative travel arrangements, could be beneficial (Currie and Muir 2017). Another option could be to improve delay repay schemes, especially since the perception of ‘value for money’ is very low for ‘class a’ (i.e., 32% satisfied, see Table 5).

The latter point highlights the final factor to which more attention should be given, the ‘value for money of the ticket price’. Table 1 suggests that only 49% of the passengers are satisfied with the ‘value for money for the ticket price’ (and only 32% for ‘class a’, 42% for ‘class b’ and 54% in ‘class c’, see Table 5). Part of this is the result of government policies, as, pre-pandemic, most railway operators relied on farebox revenue rather than subsidies (HM Government, 2016), and as a result, fares are widely considered to be expensive. Interestingly, it is mainly ‘class c’, the largest segment, that has a strong reaction to the ‘value for money of the ticket price’ attribute. Respondents in this class are more likely to travel for leisure purposes, and often make use of the cheaper tickets available on the network. More dynamic pricing could benefit this group of passengers, and there is evidence that increasing the flexibility of ticket pricing, despite making the booking experience more complex, could indeed increase demand on Britain’s railways (Anciaes et al. 2019).

Finally, policy makers and rail operators should realise that relying on traditionally defined segments, such as commuters, leisure and business travellers, does not uncover the variation observed in the data. Indeed, these segments themselves are heterogeneous, which is for instance illustrated by commuters in the data. Whilst commuters are somewhat more likely to be part of ‘class a’ and ‘class b’, many of them still have high probabilities for class c (see Table 4), which indicates that they have widely varying preferences. Further research is required to assess what causes this variation.

### Limitations

Inevitably, this study has limitations. As discussed in the data section, a data filtering procedure preceded the analysis, whereby 23,000 observations were discarded as they had one or more missing response(s) for their attribute specific satisfaction rating(s). Roughly 32,000 observations remained, which is still a very sizeable dataset. Service attributes that had more than 10% missing observations were also discarded; however, this does not mean that they are unimportant. For instance, the ‘satisfaction with connection with other train services’ was discarded due to a high number of missing values but could be very important for a specific segment of customers. Further, the independent variables were recoded into dummy variables, which improves model parsimony, but does lead to a loss of information. Finally, service attributes that were too highly correlated (i.e., correlation coefficient of more than 0.8) were discarded as well. Certain machine learning algorithms could overcome the missing value and correlation issues in future studies.

A further limitation concerns the limited set of personal characteristics that could be derived from the data (e.g., attitudes, socio-economic status), whilst measurement and inclusion of these and other factors in the class allocation model could have added value (e.g., Ye et al. 2022). Behavioural intentions, or the perceptions of alternative travel modes, such as the car, taxi or the bus, or satisfaction with individual chains of the journey are not measured in this survey. Further research into the ‘why’ behind specific satisfaction ratings, as well as research into the perception of other travel modes and trip chain specific satisfaction likely provides further information that is valuable for transport operators (Gorter et al. 2000; Abenoza et al. 2018). As discussed in the data section, there is a degree of under-sampling of commuters, which up to the COVID-19 pandemic formed the most important passenger segment on the UK railways.

Finally, since this study was performed with data gathered before the pandemic, it is unclear whether the COVID-19 pandemic will change how passengers perceive the railways, and which service attributes are most impactful. It is also unclear which passenger segments will be most important going forward, e.g., there are suggestions that the railways will be more leisure oriented in terms of its market.

## Conclusion

This study investigated the determinants of passenger satisfaction through a latent class ordered logit model while controlling for inherent unobserved heterogeneity in passenger perceptions in rail journeys. This model introduced taste variation by dividing the data into homogeneous groups, based on observed characteristics, in this case related to the trip purpose of the respondent, as well as circumstances regarding the service delivery. The results indicate that railway passengers can be segmented into three homogeneous groups, whereby significant differences in the impact of service attributes are observed between the segments. This variation would not have been uncovered through the application of traditional segmentation approaches (e.g., separate models based on observed passenger segments). One of the key findings reveals that ‘hedonic’ factors such as ‘upkeep and repair of the train’, ‘seat comfort’ and ‘value for money of the ticket price’ are most impactful on overall satisfaction for the majority of travellers, whilst ‘punctuality/reliability’ is most impactful for a small group of respondents, often on late trains, and plays a more limited role for the majority of respondents. This indicates that the role of ‘punctuality/reliability’ is more complex than assumed in the literature thus far (e.g., see Allen et al. 2019) and that merely meeting the core requirements, punctuality/reliability, is not sufficient. The findings of this study could contribute to better informed decisions facilitating the enhancement in service quality on the UK railways.

## Data Availability

The data used for this study was kindly provided by Transport Focus and the authors wish to thank Transport Focus for making the datasets available that were used. Please refer to Transport Focus to obtain the data (https://www.transportfocus.org.uk/). Data processing codes and model estimation codes are available upon request.
